# Exo- and Endo-cannabinoids in Depressive and Suicidal Behaviors

**DOI:** 10.3389/fpsyt.2021.636228

**Published:** 2021-04-23

**Authors:** Srinagesh Mannekote Thippaiah, Sloka S. Iyengar, K. Yaragudri Vinod

**Affiliations:** ^1^Valleywise Behavioral Health, Phoenix, AZ, United States; ^2^Creighton University School of Medicine, Phoenix, AZ, United States; ^3^The American Museum of Natural History, New York, NY, United States; ^4^Department of Analytical Psychopharmacology, The Nathan Kline Institute for Psychiatric Research, Orangeburg, NY, United States; ^5^Emotional Brain Institute, Nathan Kline Institute for Psychiatric Research, Orangeburg, NY, United States; ^6^Department of Child & Adolescent Psychiatry, New York University Langone Health, New York, NY, United States

**Keywords:** BDNF, HPA, CB1 receptor, depressive behavior, cannabinoids, suicide

## Abstract

Cannabis (marijuana) has been known to humans for thousands of years but its neurophysiological effects were sparsely understood until recently. Preclinical and clinical studies in the past two decades have indisputably supported the clinical proposition that the endocannabinoid system plays an important role in the etiopathogeneses of many neuropsychiatric disorders, including mood and addictive disorders. In this review, we discuss the existing knowledge of exo- and endo-cannabinoids, and role of the endocannabinoid system in depressive and suicidal behavior. A dysfunction in this system, located in brain regions such as prefrontal cortex and limbic structures is implicated in mood regulation, impulsivity and decision-making, may increase the risk of negative mood and cognition as well as suicidality. The literature discussed here also suggests that the endocannabinoid system may be a viable target for treatments of these neuropsychiatric conditions.

## Introduction

Humans have been consuming cannabis (marijuana) for more than 5000 years, and different civilizations have utilized it for varied reasons, mostly for hedonic purposes. However, in many cultures, human beings have used it to enhance religious or spiritual experiences, and for its purported medicinal value. In the middle of the 20th century, Δ^9^-tetrahydrocannabinol (Δ^9^-THC) and cannabidiol (CBD) were isolated. However, the great spur to research on endocannabinoids in the scientific community occurred when the cannabinoid-1 (CB1) receptor was cloned in 1990 (1). The discovery of the endocannabinoid system created considerable curiosity in the psychiatric research community due to its influence on neurobiological processes and neurotransmitter systems in the brain. Due to recruitment of the endocannabinoid system in reward, mood and related motivational processes (2–5), its dysfunction plays an important role in the pathophysiology of mood disorders. This review suggests that the endocannabinoid system is a viable treatment target for depressive and suicidal behavior, and discusses directions for future research.

## Exocannabinoids

**Figure 1:**
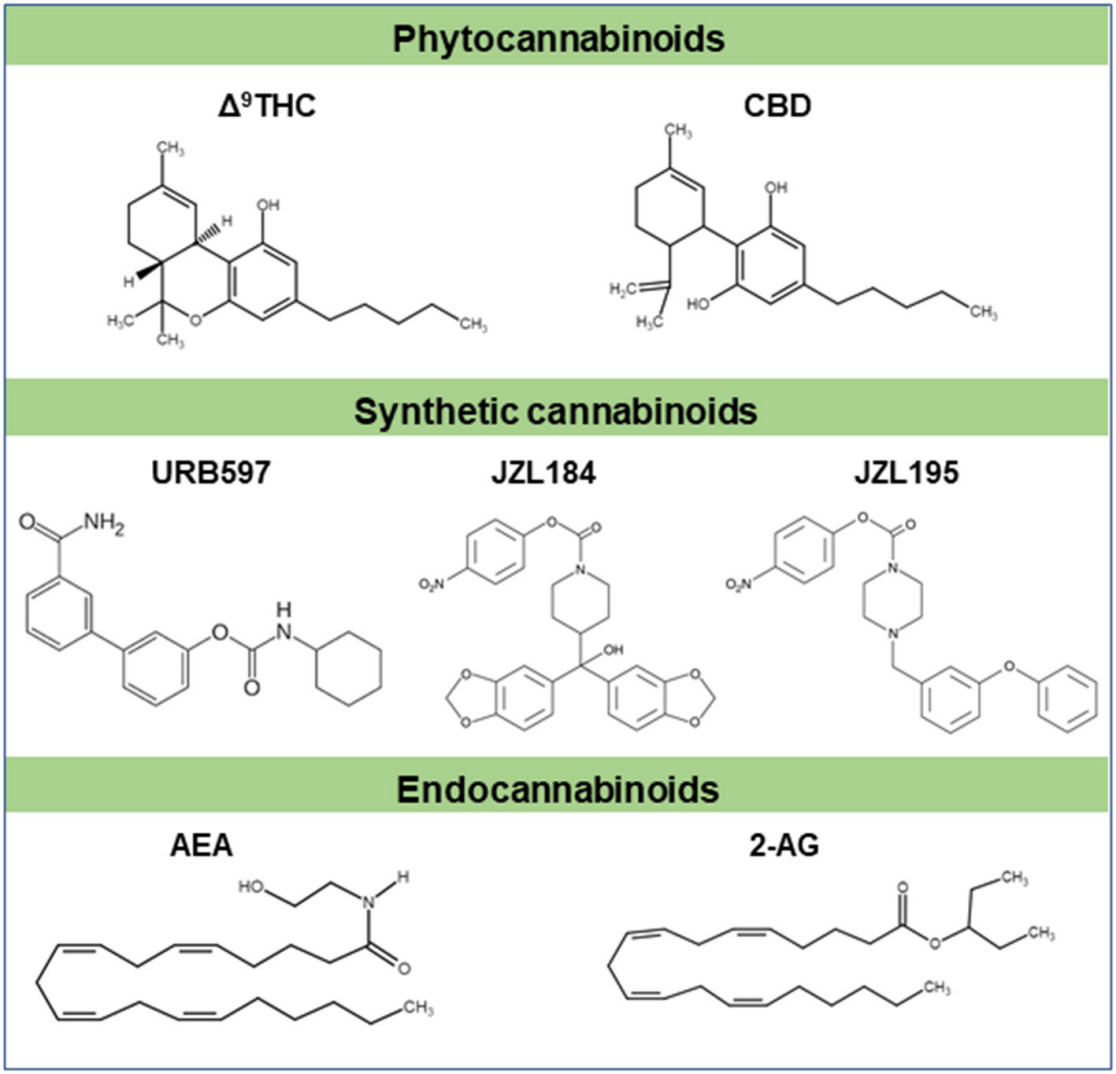
Chemical structures of some cannabinoids. Δ^9^-THC and CBD are major plant derived cannabinoids (phytocannabinoids). Synthetic cannabinoids (URB597, JZL184, JZL195 etc) are cannabimimetics. Natural cannabinoids are endogenously produced in humans and other animals are referred to as endocannabinoids (AEA, 2-AG etc). These cannabinoids typically signal through cannabinoid receptors like CB1 and/or CB2 receptors. Part of this figure has been adapted from (6) with permission.

Exocannabinoids consist of both natural and synthetic cannabinoids (Figure 1). Natural compounds of the cannabis plant are referred to as phytocannabinoids to differentiate them from the synthetic cannabinoids and endocannabinoids. In Atharvaveda, a sacred Hindu religious scripture (written between 2000 and 1400 BCE), cannabis was referred to as one of the five sacred plants, believed to be a source of happiness, bestower of joy and bringer of freedom (7). Cannabis is a genus of plants in the family “Cannabaceae” that has three common species: Cannabis Sativa, Cannabis Indica and Cannabis Ruderalis (8). Approximately 490 compounds have been identified in the cannabis plant, with more than 70 of them considered “cannabinoids” (9). The best-known and well-studied cannabinoid is Δ^9^-THC, which is the primary psychoactive constituent of cannabis. Another major compound, CBD, is a non-psychoactive substance which opposes some of the effects of Δ^9^-THC (9). Δ^9^-THC is a partial agonist at both CB1 and CB2 receptors, and its psychoactive effects are likely mediated through CB1 receptors by causing an imbalance of GABA/glutamatergic neurotransmission, as well as dopamine release. Its potential immunologic or anti-inflammatory effects are likely mediated *via* CB2 receptors (10, 11). Studies examining the effects and actions of CBD are beginning to emerge. It acts mainly through GPR55 and inhibits some of Δ^9^-THC effects *via* antagonistic/negative allosteric modulator activity at the CB1 receptor (7). It also stimulates the vanilloid receptor type 1 (VR1), similar to the effects of capsaicin, and increases arachidonoyl ethanolamide (AEA/anandamide) by inhibiting its uptake and hydrolysis (12).

Cannabis can be smoked, inhaled, mixed with food, made into snacks or drunk as a tea. The bioavailable exocannabinoids and their quantities, therefore, vary widely, depending on form and route of use. Intravenous administration and inhalation have somewhat similar pharmacokinetics and bioavailability. Δ^9^-THC and CBD are both highly lipophilic; after inhalation, peak plasma concentration reaches rapidly in few minutes (13). Δ^9^-THC has a half-life of about 6 min after the initial use, but long-term use may increase its half-life up to 22 h (14). On the other hand, CBD has a long half-life (16–30 h), and it may increase up to 5 days in daily users (14). Cannabinoids rapidly distribute into well-vascularized organs such as the lung, heart, brain, liver, and later into less vascularized organs. However, with chronic use, cannabinoids will accumulate in adipose tissues (14). Δ^9^-THC and CBD are metabolized predominantly in the liver by cytochrome enzymes (15, 16).

## Endocannabinoids

The endocannabinoid system is a neuromodulator system that consists of two classical G-protein coupled receptors (GPCRs; CB1 and CB2), their endogenous ligands (endocannabinoids) such as AEA and 2-arachidonoyl glycerol (2-AG), and the enzymes and proteins which regulate the levels of endocannabinoids (Figure 1). Recent evidence suggests that GPR55 is a purinergic non-CB1/CB2 receptor at which endo and exo-cannabinoids act, and is considered as a putative CB3 receptor (17), although it has very low homology for CB1 (14%) and CB2 (15%) (18). The CB1 receptors are expressed both in peripheral tissues and in the central nervous system. These receptors are considered to be the most abundant GPCR neuromodulatory receptors in the brain, but are also expressed in the spleen, lung, thymus, heart and vascular system (19). The activation of these receptors leads to behavioral and psychoactive effects (1). Whereas CB2 receptors are abundantly expressed in peripheral tissues such as leukocytes, spleen, tonsils, thymus, lungs and testes (20, 21). Studies also reveal the presence of CB2 receptors in the brain (22) especially in microglial cells (23–25). Using reverse transcription polymerase chain reaction, CB2 receptor mRNA expression was found in cerebellum, cortex and brainstem of the rat brain (26). GPR55 is expressed in multiple brain regions, including PFC (prefrontal cortex), amygdala and striatum (27–30), and is shown to dimerize with CB1 receptors in the striatum (31). Recent studies demonstrate that lysophosphotidyl inositol (LPI) and NAGly palmitoylethanolamide (PEA) elicit biological responses through GPR55 (18, 19, 32, 33) suggesting that these two lipids are endogenous ligands for GPR55. Some of the endocannabinoids can also activate GPR55, even though it lacks the classical CB binding pocket, and therefore it is also considered as an atypical CB receptor (34).

Human CB1 and CB2 receptors contain 472 and 360 amino acid residues, respectively. These receptors contain highly glycosylated extracellular amino-terminal and an intracellular carboxyl-terminal connected by seven transmembrane domains (20, 35). The CB1 receptors are coupled to many secondary messenger systems. They are negatively coupled to adenylyl cyclase (AC) and N- and P/Q type Ca^2+^ channels, and positively to A-type and inwardly rectifying K^+^ channels and mitogen-activated protein kinases through G_i/o_ proteins (36). The activation of CB1 and CB2 receptors leads to a reduction of cAMP production through the inhibition of AC (37). Conversely, the GPR55 is known to recruit Gα_12/13_ for signal transduction to activate phospholipase C (PLC), ERK, mitogen-activated protein kinase and Ca^2+^ release (17, 38, 39). Among endocannabinoids, AEA and 2-AG are extensively studied (Figure 1). AEA was the first one to be isolated (40), followed by 2-AG (41). There are several other endocannabinoids, such as LPI, O-arachidonoyl ethanolamine (virodhamine) (42), 2-arachidonoyl glycerol ether (noladin ether) and N-arachidonoyl-amino acids such as N-arachidonoyl dopamine (NADA) (42–46). However, the physiological role of these ligands is yet to be clearly understood. After activation of CB1 receptors on presynaptic membranes, AEA and 2-AG are degraded by enzymatic hydrolysis. The CB1 receptor-mediated retrograde regulation of synaptic strength is required to produce 2-AG whereas AEA is synthesized either during tonic control of synaptic signaling or 2-AG mediated control of synaptic strength (47).

The endocannabinoids produce varied effects due to their different affinities for the CB receptors. 2-AG is a high efficacy agonist for both CB1 and CB2 receptors whereas AEA is a low efficacy agonist at these receptors (48). These ligands are released from postsynaptic neurons in response to increase in intracellular calcium and activation of the Gq/11-linked G-protein-coupled receptors in the postsynaptic region. Upon release, they activate presynaptic CB1 and CB2 receptors, and cause transient, as well as long-lasting reduction in the release of neurotransmitters. The postsynaptic synthesis and release of endocannabinoids, and their activation of presynaptic CB1 receptors, led to the discovery of the retrograde mechanism of endocannabinoids in the modulation of neurotransmitter release from presynaptic terminals. Endocannabinoids, unlike classical neurotransmitters, are not stored in the vesicle, but they are synthesized on demand through the hydrolysis of the cell membrane lipid precursors. Several pathways for AEA synthesis have been proposed. However, AEA appears to be mainly produced from N-arachidonoyl phosphatidyl ethanol (NAPE) by the sequential action of N-acyltransferase (NAT) and N-acylphosphatidyl ethanolamine-specific phospholipase D (NAPE-PLD) (49). In immune cells and in the brain, AEA is synthesized via formation of a NAPE phosphodiester bond by a NAPE-selective PLC followed by dephosphorylation of the resulting phospho-AEA to produce AEA (50). In contrast, 2-AG is produced from 2-arachidonoyl-containing phospholipids, primarily arachidonoyl-containing phosphatidyl inositol bis-phosphate. The main biosynthetic pathway involves the hydrolysis of phosphatidylinositol by PLC, producing 1,2-diacylglycerol (DAG), which is then converted into 2-AG by a diacylglycerol lipase α/β (DAGL) (48, 51). AEA is metabolized by hydrolysis of an integral membrane protein fatty acid amide hydrolase (FAAH) into arachidonic acid and ethanolamine. The monoacylglycerol lipase (MAGL) is primarily involved in catabolism of 2-AG by hydrolysis of the ester bond between the arachidonic acid (AA) and glycerol (52, 53).

## EXO- and ENDO-Cannabinoids in Depressive Behavior

Major depressive disorder (MDD) is a debilitating disease that is characterized by depressed mood, diminished interest, impaired cognitive function, and biological symptoms such as disturbed sleep, reduced libido and appetite (54). As the 4^th^ leading cause of disability worldwide, MDD is highly prevalent (55), adversely affects the quality of life, and is significantly associated with mortality. A metanalysis of longitudinal and prospective studies in adolescents reports that use of cannabis increases the risk of developing depression and suicidal behavior in young adulthood (18 to 32 years) (56). Data from Youth Risk Behavior Survey also shows significant increase odds of reporting depression and suicidal thoughts in cannabis users (57). Although robust evidence lacks for increased risk of suicidality in acute cannabis use, chronic cannabis use has been shown to increase the risk of suicidality (58). Its use also markedly increases the risk of depression following a first episode of psychosis (59). Importantly, non-medical use of cannabis in depression was associated with higher suicidal ideation and impeded the improvement of depression (60). However, other studies found no association between depressive behavior or suicidality in cannabis use and the severe course of depressive behavior was attributed to confounding factors (61).

Stress is the most important predisposing factor for depression. Chronic stress exposure in animal models has been shown to downregulate CB1 receptor expression in several brain regions (62, 63). Male rats exposed to chronic unpredictable stress for 21 days resulted in a marked increase in CB1 receptor density as measured using radiolabeled CB1 receptor agonist, CP-55,940 in the PFC and, a reduced density in the hippocampus, hypothalamus and ventral striatum (64). These changes are accompanied with a significant reduction in anandamide levels in almost all the brain regions studied (64) suggesting an overall blunted anandamide-mediated CB1 signaling with specific alteration of the endocannabinoid system in cortical and subcortical brain regions in chronic model of depression behavior. Significant reduction of AEA and 2-AG levels have been reported due to stress but studies also show increase in 2-AG levels (63) suggesting a bidirectional regulation of these two endocannabinoids in certain brain regions. It remains to be examined whether different types and duration of psychological stressors have differential effect on other components of the endocannabinoid system.

**Figure 2:**
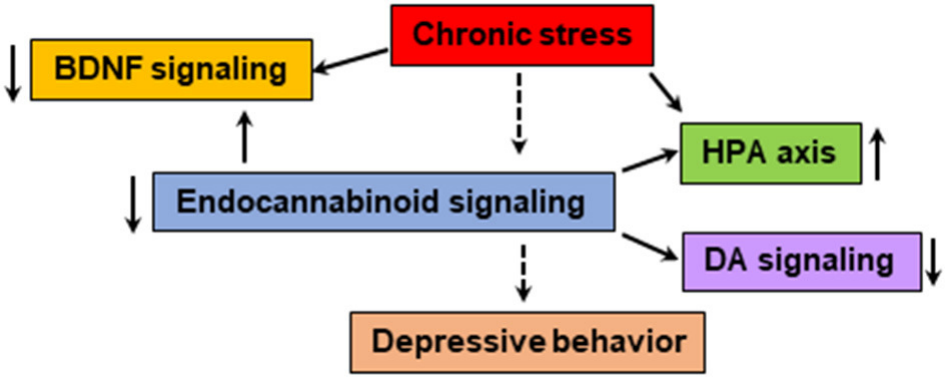
The endocannabinoid system is known to modulate the BDNF, HPA axis and monoamine neurotransmitter systems, which are shown to be impaired in patients with depressive behavior. Chronic stress could dysregulate the function of endocannabinoid-mediated signaling leading to abnormalities in neurogenesis, HPA axis, monoamine systems and immune response. The pharmacological agents that enhance endocannabinoid/CB1-mediated signaling and physical exercise shown to have positive effect on mood.

The neuroendocrine system plays an important role in the etiology and pathogenesis of mood disorders (65, 66). Chronic stress could reduce endocannabinoid signaling, leading to activation of the hypothalamic-pituitary-adrenal (HPA) axis and suppression of cell proliferation in the hippocampus (Figure 2). For instance, stress associated reduction in AEA is facilitated by corticotropin-releasing hormone (CRH) signaling could lead to an increase in AEA hydrolysis by FAAH, especially in the amygdala (11, 63, 67). This dysregulation is thought to be one of the contributing factors for anhedonia (63). Conversely, stress-induced increase in 2-AG, seen primarily in the medial prefrontal cortex (mPFC) and hippocampus, is believed to buffer and reduce the effects of stress on the brain by helping to terminate stress-induced HPA axis activation and promoting habituation to stress (63). Underscoring the role of the PFC, one suggested model entails the effect of steady levels of endocannabinoids fine-tuning GABAergic inhibition through CB1 receptors in the raphe nucleus and the basolateral amygdala. This system may be recruited in times of stress and adversity (63). These studies indicate that the endocannabinoid system plays an important role in terminating the stress response via CB1-mediated suppression of GABA release in the mPFC, likely increasing the activity of the principal neurons of the prelimbic region, which has the effect of suppressing the stress response (68). Such change in ventromedial (vm) PFC is possibly due to stress-induced increase in the levels or activity of FAAH. An increase in FAAH reduces AEA levels and this may cause a compensatory upregulation of CB1 receptor binding sites in the vmPFC in an effort to maximize the diminishing AEA signaling pool to produce mood enhancement (62). The activation of CB1 receptors has also been shown to exert antidepressant-like activity in the rat model using the forced swim test (69). This study found that the administration of AM404 (an endocannabinoid uptake inhibitor), the CB1 receptor agonist HU210 and oleamide exert similar effects as of the antidepressant desipramine. In chronic mild stress paradigm mimicking the physiological response of depression, a selective inhibitor of the FAAH enzyme, URB597 that increases AEA has been shown to exert antidepressant-like effects in Wister rats (70) but such effects were not reported in standard test condition through pharmacological inhibition or genetic deletion of FAAH. However, when methodological changes were made such as using altered ambient light, significant effects on emotional reactivity were observed (71). Taken together enhancement of AEA-mediated signaling appears to reduce depressive behavior (72, 73).

In women with major depression, serum 2-AG levels correlated with the duration of the depressive episode: the longer the duration of the depression, the lower the 2-AG levels. Such changes were not seen in regard to AEA but a strong negative correlation was observed with serum AEA levels and anxiety symptoms in affective disorders (74). A subsequent study found that basal serum concentrations of AEA and 2-AG were significantly reduced in women with major depression (75). Serum 2-AG but not AEA levels were found to increase after the social stress test in the same patients (75). Interestingly, serum levels of other endocannabinoid ligands such as PEA and OEA were also lower in these patients (75). An increase in serum AEA levels with a corresponding reduction in depressive symptoms has also been shown in women with MDD after moderate exercise (76). Though exercise can improve mood through other signaling mechanisms such as serotonin (5-HT) (77), varying levels of aerobic exercise increases AEA and 2-AG (78). Additionally, a 30 min exercise as an adjunctive treatment in substance use disorder elevates AEA level and improves mood (79). Interestingly, exercise increases not only AEA and 2-AG but also lipid messengers OEA, PEA, N-docosahexaenoyl ethanolamine and 2-oleoylglycerol (80) resulting in an acute improvement in pain and mood (81). Moreover, exercise-induced increase in AEA levels elevates BDNF, and influences neuroplasticity and the antidepressant effects (82). Such mood-enhancing properties of endocannabinoids may also play a role in non-suicidal self-injurious behavior, a negative behavior that could reduce the intensity of negative affective states (83). It is important to note that circulating endocannabinoids can be derived from multiple organs, tissues, immune cells etc. (46), and thus the association between endocannabinoid levels in blood and brain is currently not clear. Nevertheless, an intravenous administration of endocannabinoids has been shown to enhance reward-seeking behavior in rats (84, 85) suggesting that circulating endocannabinoids might have central effects to some degree as these lipids can readily cross the blood brain barrier (86) and activate reward and other neuronal processes.

It is well-documented that women are more susceptible to MDD than men (54, 87–89). There have been some attempts to delineate biological causes of this dimorphism, but reasons remain obscure. Studies in animal models have shown that chronic stress could dysregulate the endocannabinoid system differently in males and females. For instance, chronic stress significantly downregulates CB1 receptor levels in the hippocampus of male rats, while it upregulates these receptors in the dorsal hippocampus of female rats (90, 91). Our recent study revealed a decrease in postmortem levels of AEA and 2-AG in the ventral striatum (nucleus accumbens; NAc) of women with MDD as well as in female Wistar Kyoto (WKY) rat, a genetic model of depression (92). This study also found lower levels of BDNF in the ventral striatum of MDD patients. Interestingly, pharmacological inhibition (by use of JZL195) of the two major endocannabinoid degrading enzymes, FAAH and MAGL, elevated both endocannabinoids and BDNF levels in the ventral striatum, and reduced depressive-like behavior in female rats (92). The ventral striatum has been shown to play a central role in reward and motivation related processes that become dysfunctional in mood disorders (93, 94). The deficiency in endocannabinoid-mediated signaling in this brain region may impact reward processing leading to anhedonia, a major symptom of clinical depression. Besides BDNF, dopamine signaling in the ventral striatum plays a critical role in the pathogenesis of MDD (94–96). Both AEA and 2-AG, as well as pharmacological activation of CB1 receptors, elevate dopamine release in the NAc (97, 98) and increase hedonic taste (97). The behavioral effects of JZL195 are most likely associated with endocannabinoid-mediated activation of dopaminergic signaling in the ventral striatum which promotes reward and motivation-related processes. Beside reward deficit, social withdrawal is one of the major symptoms observed in patients with MDD. Recent studies suggest that the endocannabinoid system mediates social behavior in rodent models. Systemic administration of JZL195 has been shown to enhance social interaction in WKY rats (92). The deletion of DAGLα (2-AG synthesizing enzyme) in direct medium spiny neurons of the striatum elicits deficiency in social behavior in mouse (99). Moreover, pharmacological augmentation of 2-AG via administration of JZL184 (MAGL inhibitor), reduces glutamatergic activity at basolateral amygdala-NAc synapse and rescues deficits in social interaction (100). These studies underscore the importance of endocannabinoids in regulating social behavior.

The CB1 agonists and FAAH inhibitors can also enhance central 5-HT and noradrenergic (NE) transmission and promote neurogenesis in the hippocampus (101). Such effects are also observed with most of the currently available antidepressants. In this regard, FAAH inhibitors may have more beneficial effects than CB1 agonists due to the lack of adverse cannabinoid side-effects and a better therapeutic window (101). The antidepressant-like effects of CBD have been shown in genetic animal model of depression (102) suggesting that CBD is a potential for the treatment of clinical depression and anhedonia. Conversely, besides some beneficial effects in certain illnesses like pain and spasticity (103), no strong favorable effects of Δ^9^-THC in depressive behavior have been reported. Δ^9^-THC can also have short and long-term adverse effects (104, 105). The studies which evaluated the effects of cannabinoids for pain have found no significant difference between cannabinoids such as dronabinol (Δ^9^-THC) and nabiximol (Δ^9^-THC and CBD) in improving depression compared to placebo when depression was used as an outcome measure (105–107). In fact, nabiximol (Δ^9^-THC and CBD) had negative effects on depressive symptoms at higher dose but no difference in improving depression when compared to placebo at lower doses (106). Further detailed studies on use of cannabis products for treating mental illnesses are clearly needed due to lack of well-designed randomized trials and small sample size (108).

## The Endocannabinoid System in Suicide and Impulsivity

Suicide accounts for 1.4% of all deaths worldwide (109). About 30% of individuals with mood disorders die by suicide (110). Up to 87% of suicide victims suffer from major depression, while up to 15% of patients with unipolar depression are most likely to commit suicide (111). When depression is comorbid with alcohol use, the suicide rate increases significantly (112). Impulsive behavior is also an important risk factor for suicide (113). A study conducted in combat veterans with and without a history of suicide attempts observed a higher serum concentration of 2-AG among suicide attempters; in addition, stress-induced cortisol levels positively correlated with 2-AG levels (114). However, AEA levels negatively correlated with suicide ideation scores among attempters (114). Another study that examined victims of the 9/11 World Trade Center disaster found a positive correlation of AEA levels with circulating cortisol (115).

The PFC is necessary for healthy neurocognitive function and the pathogenesis of depression correlates with relative hyperactivity in vmPFC and hypoactivity in the dorsolateral PFC (dlPFC) (116). Another cortical area, the dlPFC plays a primary role in cognitive functions such as working memory, goal-directed action, abstract reasoning and judgment. These executive functions are significantly affected in depression (117) and suicidality. Increased activity of vmPFC plays an essential role in the generation of negative emotion. Lesions in the vmPFC can cause a loss of self-awareness and insight, with a marked reduction in feelings of shame, guilt, embarrassment, and regret (117). Abnormal activation of the orbitofrontal prefrontal cortex is also shown to increase compulsivity and repetitive behaviors, similar to the behaviors seen in substance use disorders (118). The multiple domains of PFC functional impairment manifest as impaired cognitive control of mood, pessimism, impaired problem solving, over-reactivity to negative social signs, excessive emotional pain, and suicidal ideation (119). These effects cumulatively contribute to an increased risk of suicide. A localized PFC hypofunction in people who have attempted suicide was proportional to the lethality of the suicide attempt (120).

A postmortem study of patients with MDD who committed suicide revealed a higher density of CB1 receptors in the dlPFC (121). Whether or not this elevation in CB1 receptors is due to neuroadaptation to lower levels of endocannabinoid is currently unknown. Both the activity and level of the FAAH enzyme (which degrades AEA) are found to be lower in the ventral striatum of individuals with alcohol use disorder (AUD) when compared to non-psychiatric controls (122). In suicide victims with AUD, the level and activity of FAAH are significantly higher compared to the group with AUD who did not commit suicide (122). These observations suggest that suicide may be associated with upregulation of CB1 receptors in the ventral striatum. While higher levels of endocannabinoids were also found in suicide victims in this study, additional well-characterized postmortem samples are needed to tease out this finding due to potential confounding effect of alcohol use on endocannabinoid system in these subjects. The positive correlation of upregulation of CB1 and FAAH activity indicates that CB1 receptor sensitization in the ventral striatum of suicide victims could be contributed to a decrease in AEA levels. However, an increase in CB1 receptor expression and lower levels of MAGL activity were reported in postmortem PFC of subjects with AUD (123). In addition to the findings related to CB1, density of CB2 receptors and GPR55 gene expression were found to be significantly lower in the dlPFC of suicide victims (124). Nevertheless, higher protein levels of CB2 receptors in both neurons and astrocytes were observed in the dlPFC of suicide victims (124). This suggests that CB2 receptors and GPR55-mediated signaling mechanisms may also play a role in the pathophysiology of suicidal behavior. These findings may have etiologic and therapeutic implications for the treatment of suicidal behavior. In addition to mood disorders, substance use disorders, including alcohol are independently associated with an increase in the risk of suicide (125–127). In this regard, a postmortem study revealed elevated levels of CB1 receptors in the dlPFC of patients with AUD who were suicide victims, compared with patients with AUD who were not victims of suicide (128). This finding that resembles the findings in depressed suicide victims (121), supports the evidence linking sensitization of cortical CB1 receptors to suicide. Higher levels of the endocannabinoids AEA and 2-AG were also observed in the dlPFC of suicide victims who diagnosed with AUD (128) suggesting an endocannabinoid system dysregulation in the PFC of suicide victims.

## Conclusion and Future Perspectives

The endocannabinoid system and its role in psychiatric disorders is a rapidly growing area of research. The evidence discussed in this review supports that the endocannabinoid system is an integral part of the neurobiological processes that regulate reward, stress response and mood. Dysfunction of the endocannabinoid system could contribute to the manifestation of behavioral abnormalities seen in depression and suicidal behavior. It is important to note that depressive disorder comprises a cluster of complex symptoms which has a heterogeneous presentation across patients. Although dysregulation of the 5-HT and NE systems are implicated in the pathophysiology of mood (129), many patients do not adequately respond to existing antidepressants which are targeted to these neurotransmitter systems. While the endocannabinoid system modulates the function of HPA axis, neurotrophic factor (BDNF), and many neurotransmitter systems, including 5-HT, NE and DA, a dysfunction in endocannabinoid system most likely has a greater effect on the functions of these systems and mood related behaviors (Figure 2). It is difficult to delineate whether any symptoms specific to dysregulation of the endocannabinoid system or the monoaminergic system predispose to depressive and suicidal behaviors. Thus, mere enhancement or modulation of the endocannabinoid system may not be the best treatment modality. Perhaps therapeutic agents which target one or more of these systems simultaneously might be the goal for future research. There are no conclusive findings until now which clearly demonstrate specific modulatory effects of the endocannabinoid system on suicidal thoughts.

There are inconsistent findings on the role of cannabis in depressive and suicidal behaviors which stem from confounding factors. The reported pharmacologic effects targeting endocannabinoid system also remain somewhat inconsistent, due to lack of selective reagents and use of diverse behavioral paradigms in preclinical studies. Current, advanced neuroscientific techniques can improve understanding of the significance of endocannabinoid signaling, including its effects on regulation of mood and reward processes. New therapeutic agents that act on the endocannabinoid system are most likely to emerge as pharmacologic treatment options for depression and many other disorders. The pharmacologic agents which are available for use in clinical practice need further research into their safety and therapeutic benefits. In addition to the pharmacological strategies, physical exercise is beneficial in elevating the endocannabinoids and mood. Whether circulating endocannabinoids could serve as biomarkers for the diagnosis and prognosis of MDD and other psychiatric disorders need to be determined. It remains to be examined if depressive and suicidal behaviors are independently related to dysfunction in specific components of the endocannabinoid system. The mechanisms driving the gender and brain region-specific changes seen in these disorders remain unclear, and further studies are warranted in this field.

## Author Contributions

All the authors have significantly contributed to the development of this review article.

## Conflict of Interest

The authors declare that the research was conducted in the absence of any commercial or financial relationships that could be construed as a potential conflict of interest.
